# Volatile components and nutritional qualities of *Viscum articulatum* Burm.f. parasitic on ancient tea trees

**DOI:** 10.1002/fsn3.1159

**Published:** 2019-08-11

**Authors:** Qiushuang Wang, Dong Chen, Qianwen Zhang, Dandan Qin, Xiaohui Jiang, Hongjian Li, Kaixing Fang, Junxi Cao, Hualing Wu

**Affiliations:** ^1^ Tea Research Institute, Guangdong Academy of Agricultural Sciences Guangdong Key Laboratory of Tea Plant Resources Innovation & Utilization Guangzhou China; ^2^ Department of Plant and Soil Sciences Mississippi State University Starkville MS USA

**Keywords:** aroma, nutrients, *Viscum articulatum* Burm.f.

## Abstract

Volatile flavor compounds (VFCs) and nutrients in *Viscum articulatum* Burm.f. parasitic on ancient tea trees (named TM) were studied in this research by headspace solid‐phase microextraction (HS‐SPME)/gas chromatography–mass spectrometry (GC–MS) and conventional methods. Sixty‐six volatile compounds belonging to different classes were identified by GC–MS. The ketones, alcohols, and aldehydes were the principal aroma groups in TM according to principle component analysis (PCA). The most abundant aroma components in TM included benzaldehyde (9.64%), geranylacetone (7.92%), epoxy‐*β*‐ionone (7.71%), *β*‐linalool (7.35%), methyl salicylate (6.96%), and hotrienol (6.14%), significantly higher than CKs (*p* < .05). The positive PC1 and PC2 in TM were correlated with benzaldehyde, hotrienol, methyl salicylate, and geranylacetone. The mistletoes could be differentiated from CKs due to the difference in aroma compounds. Clean and fresh, woody and nutty odor with minor floral scent was the characteristics of TM. Analysis of the nutritional components showed that contents of polyphenols and catechins in TM were at trace levels, significantly lower than CKs (*p* < .05). The total contents of polyphenols, amino acids, carbohydrates, and caffeine in TM were significantly lower from the total soluble solids (*p* < .05), indicating that there were still lots of compounds undetected in TM. The sensory test showed that the taste and aroma in TM can be accepted, which indicates TM could be developed into alternative tea drinks in the future.

## 1 INTRODUCTION

**Figure 1:**
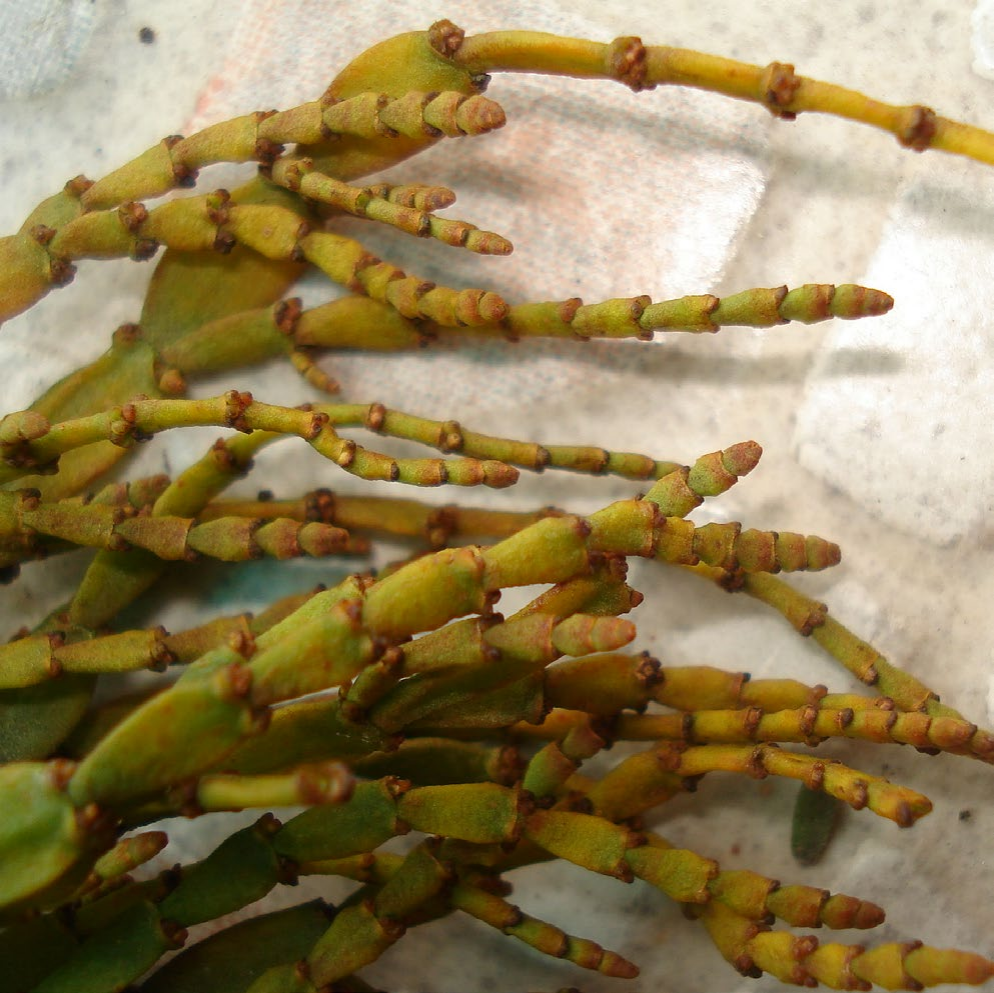
Fresh mistletoe hosted in ancient tea trees

*Viscum articulatum* Burm.f. (*V. articulatum*), a parasitic herbaceous perennial plant, is commonly hosted in oak trees and other deciduous trees that are upwards of thousands of years old (Choi et al., 2008; Sun, Si, Liu, & Zhang, 2012). *Viscum articulatum* hosted in ancient tea trees is named tea mistletoe (TM) and is known as “Pangxiejiao” in Chinese, due to its coral shape, short branches, and hair, similar to crab's legs (Sun et al., 2012) (Figure 1). TM and the famous traditional Chinese medicine *Viscum coloratum* (Kom.), Nakai, both belong to genera in the Loranthaceae (Zhao et al., 2011), which contains special biological components, such as flavanols, flavonoids, alkaloids, organic acids, and other high molecular weight compounds (Ni, Li, Zhao, Shao, & Li, 2013). Experiments have been conducted to attest their potential health activities, such as antibacterial, antimutagenic, anti‐arrhythmia effects, immunomodulating effects, eliminating rheumatalgia, curing knee–waist malaise and hepatitis (Bachhav, Bhutada, Patil, Baser, & Chaudhari, 2012; Lee, Kim, Kim, Park, & Lee, 2009; Yang, Xiang, Feng, & Liu, 2009). Therefore, TM becomes a popular drink among local residents, which has been developed into commercial products for sale by tea companies. When soaked, TM releases a special fresh aroma, which is significantly different from teas. However, there is little information in the literature about the aroma characteristics and nutrients of TM. It is also worth studying whether TM has similar nutritional ingredients to teas because of their parasitism.

The various aromas of teas such as fruity‐sweet, floral‐sweet, and fresh grass present an attractive odor organoleptic quality. Tea aroma is an important quality parameter and main sensory properties for quality of teas (Wang, Chen, Xu, & Yin, 2012; Yang, Baldermann, & Watanabe, 2013). It is important to identify the key aroma compounds of teas with different aroma types, which could increase the understanding of the basic theory of tea aroma chemistry. Extraction was the first step to analyze the aroma. Methods to extract aroma compounds included simultaneous distillation extraction (SDE) (Rawat et al., 2007), hydrodistillation, HS‐SPME, and supercritical‐fluid extraction (SFE) (Bonino et al., 2003; Mounchili et al., 2005). Among them, HS‐SPME has been proven to be an effective isolation and concentration technique for volatile compounds (Li, Lee, & Shen, 2006; Lv et al., 2012; Monje, Privat, Gastine, & Nepveu, 2002; Mounchili et al., 2005). It has the advantage of being simple, sensitive, solvent‐free, time‐ and labor‐saving (Wang et al., 2008), and low boiling point volatile preservation (Lv et al., 2012) with no need for concentration in comparison with other extraction methods (Huang et al., 2011). Therefore, this method has been applied in food, medical, biological, and environmental fields (Cuevas‐Glory, Pino, Santiago, & Sauri‐Duch., 2007; Guffanti, Pifferi, Falciola, & Ferrante, 2018; Hansen, Frandsen, & Fromberg, 2016).

In this study, HS‐SPME/GC–MS methods were used to analyze the aroma of TM, oak mistletoe (OM), and the control samples. The nutritional qualities including the polyphenols, catechins, caffeine, and soluble carbohydrates were analyzed by HPLC and spectrophotometry methods. The objectives of our research were to reveal the aroma profile of TM as well as its nutritional compounds in order to provide scientific foundation for its deeper development.

## 2 MATERIALS AND METHODS

### 2.1 Chemicals

Caffeine, *β*‐glucosidase, standard catechins ((−)‐epigallocatechin (EGC), (−)‐epicatechin (EC), (−)‐epicatechin gallate (ECG), (−)‐epigallocatechin gallate (EGCG), (−)‐gallocatechin (GC), and (−)‐gallocatechin gallate (GCG), etc.) were purchased from Sigma‐Aldrich China‐Mainland (Shanghai, China). Other chemicals, such as methanol, acetic acid, acetonitrile, sulfuric acid, FeSO_4_, glutamic acid, *β*‐glucosidase, and anthrone, were of analytical grade or of the highest purity available.

### 2.2 Materials

One TM sample, one mistletoe hosted in oak trees (OM), and two control tea samples (CK1 and CK2) were provided by Tea Research Institute, Guangdong Academy of Agricultural Sciences in May, 2016. The TM sample was collected from old, former tea trees no longer in production. The control tea samples were collected from local tea garden. All the samples were dried, ground, sieved with a 40 mm (aperture) sieve, and stored in airtight glass containers at 4°C for further use. The details of samples were shown in Table 1.

**Table 1:** Sample details in this research

Sample name	Details	Tenderness
Tea mistletoe (TM)	Hosted in ancient tea trees	One stem with one to two branches
Oak mistletoe (OM)	Hosted in ancient oak trees	One stem with one to two branches
Steamed green tea (CK1)	Fresh tea leaf from local tea trees, dried	One bud with two leaves
Pureh raw tea (CK2)	Fresh tea leaf from local tea garden, made into Pu‐erh raw tea	One bud with two leaves

### 2.3 Analysis of aroma components

#### 2.3.1 HS‐SPME procedure

The HS‐SPME method was used to extract the VFCs from experimental samples. The HS‐SPME holder was fiber‐coated with Divinylbenzene/Carboxen/Polydimethylsiloxane (DVB/CAR/PDMS) at a thickness of 50/30 μm (Supelco Co.). As indicated by the instruction manual, the fiber was activated for 5 min in the GC injection port to eliminate impurities before GC analysis.

The VFCs were extracted according to the method presented by Lv et al. (2012) with slight modification. Of 10.0 g of tea powder was weighed and placed in a 250 ml conical flask and immediately infused with 100 ml of boiling water. A magnetic heating stirrer was used to establish equilibrium faster at the temperature of 50°C. The SPME fiber was exposed in the headspace of the conical flask for 80 min and then introduced into the GC injection port to allow desorption for 3.5 min.

#### 2.3.2 GC–MS analysis

The extracted VFCs from samples were analyzed on a GC–MS (TRACE DSQ, Thermo Finnigan). GC conditions were as follows: injector temperature, 230°C; injector mode, splitless mode; column, HP‐5MS, 30 m, 0.25 mm i.d., 0.25 µm film thickness. The GC oven temperature was programmed to hold at 50°C for 1 min, then increase to 180°C at a rate of 2°C/min for 1 min; and finally to 230°C at a rate of 10°C/min, which was maintained for another 1 min. Helium (percentage purity > 99.999%) was used as the carrier gas at a constant flow velocity of 1 ml/min.

MS spectrometer conditions were as follows: interface temperature, 280°C; ion source temperature, 220°C; ionization mode, EI; electron energy, 70 eV; mass scan range, 40–400 AMU. The National Institute of Standards and Technology (NIST) library (98 L), MS, retention time compared with those reported in the literature were used to identify aroma compounds.

The relative proportions of the constituents were obtained by FID peak area normalization and were quantified by the following equation:

Relative content % = single constituent area/total area × 100%

The internal standard was not added in this research. Because the internal standard changed the equilibrium system of tea soup and would also be absorbed by the teas, it was feasible to use the method of relative peak area to analyze and compare the aroma components of the different samples in the same experimental conditions.

### 2.4 Analysis of major nutritional constituents

#### 2.4.1 The total soluble solids

The total soluble solids were determined according to the method of Yin, Xu, Yuan, Luo, and Qian (2009), with minor modifications. The ground samples (1.0 g) were extracted in 100 ml of boiled distilled water for 45 min. The extracted infusion (50 ml) was placed in a weighed evaporation dish to evaporate to dryness on a heated water bath. Then, the evaporation dish was dried at 120°C for 2 hr until the weight of the dishes with the residues reached to a constant content. The contents of soluble solids were calculated according to the weight difference of the evaporation dishes before and after evaporation.

#### 2.4.2 Analysis of tea polyphenols

Tea polyphenols were determined with spectrophotometric method using FeSO_4_, potassium sodium tartrate and phosphate of pH7.5 as a buffer (Xu et al., 2018). Of 1.0 g samples was extracted with 100 ml of boiled distilled water for 45 min. Of 1.0 ml of the above infusion, 4.0 ml of distilled water, 5.0 ml of dyeing solution, and 15 ml of buffer was mixed to determine the absorbance (A1) at 540 nm using a 1‐cm photometer cuvette and a UV/VIS 752N spectrophotometer (Shanghai, China). The absorbance (A2) of the control was determined in the same conditions, in which the extracted infusion was replaced by distilled water. Polyphenols content (%) = [(A1 − A2) × 3.91 × V] × 100/(m × 10^3^), where (A1 − A2) was the difference between two absorbance, m was the dry weight of samples, g, V was the total volume of infusion, ml.

#### 2.4.3 Analysis of catechins and caffeine

Catechins and caffeine were analyzed with HPLC method (Xu et al., 2015). Of 0.2 g tea powder was extracted in 70% methanol for two times. And then, the extract infusions were filtered through a 0.22‐μm Millipore filter before injection (Model Agilent 1200 E, Agilent). The HPLC conditions were as follows: injection volume: 10 µl; column: 5 μm ZORBAX Elips XDB‐C18 (4.6 × 150 mm); temperature: 30°C; mobile phase A: acetonitrile/acetic acid/methanol/water (2:1:4:193); mobile phase B: acetonitrile/acetic acid/methanol/water (20:1:40:127); gradient: 100% mobile phase A to 100% mobile phase B by linear gradient during the first 25 min and then 100% mobile phase B for 5 min; flow rate: 1 ml/min; detector: Agilent SPD‐10A ultraviolet detector (Agilent Corporation) at 280 nm. Contents of catechins and caffeine were calculated according to the area normalization method.

#### 2.4.4 Analysis of free amino acids

The content of amino acids was determined according to the ninhydrin dyeing method at 570 nm using glutamic acid as the standard for the amino acids (Yao, Guo, Lu, & Jiang, 2005). Of 1.0 ml of tea infusion with 0.5 ml of ninhydrin solution (2%) and 0.5 ml of phosphate buffer (pH = 8.0) was heated in water bath for 15 min before cooling and fixing to 25 ml with water. The absorbance (A) was determined at 570 nm with a spectrophotometer. The amino acids content (%) = (C × V_1_)/(10 × V_2_ × M × W), where C was the quality of glutamic acid calculated from the standard curve based on the absorbance value (A), mg; V_1_ was the total volume of the infusion, ml; V_2_ was the volume of test solution, ml; M was sample mass, g; W was the dry matter content, %.

#### 2.4.5 Analysis of soluble carbohydrates

Soluble carbohydrates were analyzed by anthrone–sulfuric acid reaction, using glucose as a standard. Tea infusion was made as described in 2.4.2. Of 1.0 ml of tea infusion was reacted with 8.0 ml of anthrone reagent (1.0 mg/ml anthrone–pure sulfuric acid solution) at 100°C for 7 min, and then, the absorbance (A) was determined at 620 nm after the mixture was cooled rapidly for 10 min. The soluble carbohydrate content (%) = C × V_1_/M × m × 10, where C was the concentration of glucose, calculated from the standard curve based on the absorbance value (A), mg; V_1_ was the volume of solution, ml; M × m was the dry weight of the sample, g.

### 2.5 Sensory evaluation

Sensory tests were conducted by five panelists in Guangdong Key Laboratory of Tea Plant Resources Innovation & Utilization, mainly based on the outer shape, the residues, the aroma, and the taste of the infusions, according to National Standard of the People's Republic of China (Gong et al., 2018).

### 2.6 Statistical analysis

Significance test was carried out using SPSS software (version 16.0; SPSS, Inc.), where one‐way analysis of variance (ANOVA) and the Tukey test were used (a *p*‐value of .05 was considered significant). PCA was carried out using Excel 2019 supported by XLSTAT software to determine the relationships between samples and volatile aroma compounds. All the experiments were repeated three times under identical conditions. Data were expressed as the average of three replications.

## 3 RESULTS AND DISCUSSION

### 3.1 Identification and quantification of the VFCs

A total of 66 volatile compounds, including 12 ketones, 12 aldehydes, 12 alcohols, 11 esters, 5 alkene, 5 hydrocarbons, 2 acids, 2 phenolic compounds, and 5 other compounds were identified by GC–MS in this study (Table 2). They accounted for 32.45%, 20.38%, 16.92%, 18.60%, 0.51%, 6.58%, 3.12%, 1.14%, and 0.30% of the total aroma in TM.

**Table 2:** Volatile compounds identified in this research using GC–MS

Peak no.	Compounds	Retention time (min)	TM (%)	OM (%)	CK1 (%)	CK2 (%)	Odor note
Alcohols							
1	1‐Octene‐3‐ol	18.91	–	–	0.30 ± 0.02	0.94 ± 0.08	Mushroom^a^
2	Phenylethyl alcohol	22.03	0.10 ± 0.00	13.08 ± 0.29	0.48 ± 0.04	0.28 ± 0.03	Sweet, flower^b^
3	*cis*, Linalool oxide	24.68	–	0.02 ± 0.00	0.45 ± 0.03	0.93 ± 0.05	Flower, wood^a^
4	*β*‐Linalool	25.36	7.35 ± 0.21	10.41 ± 0.25	37.39 ± 1.83	48.60 ± 2.13	Flower, lavender, wood^a^
5	Hotrienol	25.62	6.14 ± 0.14	5.78 ± 0.24	5.49 ± 0.25	4.33 ± 0.19	Hyacinth^a^
6	*α*‐Terpineol	30.31	0.01 ± 0.00	0.01 ± 0.00	2.27 ± 0.24	1.72 ± 0.22	oil, anise, mint^a^
7	Geraniol	31.66	2.10 ± 0.13	2.69 ± 0.11	9.17 ± 0.12	8.41 ± 0.23	Sweet, rose^a^
8	*β*‐Ionol	41.41	0.02 ± 0.07	0.01 ± 0.00	0.28 ± 0.05	0.25 ± 0.07	n.d.^c^
9	*E*‐Nerolidol	46.50	0.60 ± 0.04	1.08 ± 0.09	1.32 ± 0.06	0.95 ± 0.04	Wood, flower, wax^a^
10	*α*‐Cedrol	48.77	0.60 ± 0.03	0.71 ± 0.04	0.97 ± 0.04	0.33 ± 0.06	Wood^a^
11	*α*‐Cadinol	49.99	–	–	0.68 ± 0.03	0.34 ± 0.08	Herb, wood^a^
12	Phytol	60.12	–	–	3.16 ± 0.14	2.76 ± 0.21	Flower^a^
Aldehyde							
13	2‐Heptenal	17.65	0.06 ± 0.01	0.18 ± 0.04	–	0.10 ± 0.01	Soap, fat, almond^a^
14	Benzaldehyde	17.97	9.64 ± 0.22	14.20 ± 0.26	1.47 ± 0.09	0.84 ± 0.07	Almond, burnt sugar^a^
15	Octanal	20.24	1.34 ± 0.09	0.49 ± 0.04	0.11 ± 0.03	0.10 ± 0.02	Fat, soap, lemon, green^a^
16	2,4‐Heptadienal, (E,E)‐	20.64	0.75 ± 0.12	0.99 ± 0.08	0.01 ± 0.00	0.29 ± 0.01	Fried^a^
17	Benzyl acetaldehyde	22.48	0.01 ± 0.00	0.01 ± 0.00	0.74 ± 0.10	1.73 ± 0.11	Hawthorne, honey, sweet^a^
18	2‐Octenal, (E)‐	23.16	0.56 ± 0.07	0.85 ± 0.06	0.33 ± 0.04	0.35 ± 0.09	Green, nut, fat a
19	*Cis*‐2‐Nonenal	28.43	0.42 ± 0.10	0.46 ± 0.03	0.13 ± 0.02	0.26 ± 0.03	Orris, fat, cucumber^a^
20	Safranal	30.52	1.03 ± 0.05	1.47 ± 0.17	0.23 ± 0.02	0.63 ± 0.08	Herb, sweet^a^
21	Z‐Decanal	30.76	1.48 ± 0.08	1.21 ± 0.05	1.03 ± 0.04	0.46 ± 0.05	Soap, orange peel, tallow^a^
22	*β*‐Cyclocitral	31.51	2.31 ± 0.24	1.02 ± 0.07	0.87 ± 0.04	1.70 ± 0.06	Mint^a^
23	(*E*)‐2‐Decenal	33.47	2.11 ± 0.09	1.84 ± 0.11	0.28 ± 0.08	0.29 ± 0.05	Tallow^a^
24	*cis*‐ Citral	33.72	0.67 ± 0.04	0.33 ± 0.03	0.61 ± 0.08	0.69 ± 0.05	lemon^a^
Ketones							
25	Nonanone	24.86	1.55 ± 0.16	0.58 ± 0.05	–	–	Hot milk, soap, green^a^
26	6‐Methyl‐3,5‐heptadien‐2‐one	25.51	1.78 ± 0.13	0.01 ± 0.00	–	–	n.d.^c^
27	2‐Decanone	30.02	1.89 ± 0.15	‐	–	–	Fruit^a^
28	2‐Undecanone	34.87	0.67 ± 0.05	0.41 ± 0.03	–	–	Orange, fresh, green^a^
29	*cis*‐Jasmone	39.53	–	–	2.32 ± 0.06	0.95 ± 0.26	flower^f^
30	6,10‐Dimethyl‐2‐undecanone	39.84	0.89 ± 0.06	0.15 ± 0.02	0.08 ± 0.02	0.06 ± 0.01	n.d.^c^
31	*α*‐Ionone	40.77	1.83 ± 0.16	1.64 ± 0.18	2.66 ± 0.21	1.37 ± 0.11	Wood, violet a
32	*β*‐Dihydrogen ionone	41.28	0.62 ± 0.06	1.70 ± 0.13	–	–	n.d.^c^
33	Geranylacetone	41.93	7.92 ± 0.23	4.19 ± 0.12	2.06 ± 0.12	1.75 ± 0.16	Magnolia, green, fruit^a^
34	*β*‐Ionone	43.20	4.10 ± 0.19	3.92 ± 0.12	3.40 ± 0.17	3.82 ± 0.23	Seaweed, violet, flower^a^
35	Epoxy‐*β*‐ionone	43.37	7.71 ± 0.21	2.33 ± 0.20	1.24 ± 0.07	0.65 ± 0.03	Fruit, sweet, wood^a^
36	2‐Pentadecanone, 6,10,14‐trimethyl	55.52	3.49 ± 0.25	1.51 ± 0.10	0.25 ± 0.03	0.34 ± 0.03	Fat^a^
Esters							
37	*Trans*‐3‐Hexenyl butyrate	29.66	0.01 ± 0.00	‐	0.49 ± 0.04	0.12 ± 0.01	n.d.^c^
38	Methyl salicylate	30.16	6.96 ± 0.24	4.79 ± 0.17	4.13 ± 0.48	4.46 ± 0.25	Peppermint^a^
39	*Cis*‐3‐Hexenyl isovalerate	32.15	2.88 ± 0.34	0.61 ± 0.04	0.18 ± 0.04	0.14 ± 0.03	n.d.^c^
40	*γ*‐Nonanolide	38.00	3.69 ± 0.15	0.15 ± 0.03	–	–	Coconut fragrance, fruit aroma^d^
41	Nerol acetate	38.75	–	0.07 ± 0.00	0.26 ± 0.02	0.39 ± 0.07	Orange^a^
42	*cis*‐Hexanoic Acid, 3‐hexenyl ester	38.83	0.64 ± 0.08	0.33 ± 0.02	3.85 ± 0.21	0.53 ± 0.04	n.d.^c^
43	*Trans*‐2‐Hexenyl caproate	39.20	0.70 ± 0.02	0.01 ± 0.00	0.20 ± 0.01	0.12 ± 0.01	n.d.^c^
44	Dihydro actinidiolide	45.39	2.77 ± 0.24	2.77 ± 0.21	0.32 ± 0.04	0.40 ± 0.05	Woody^e^
45	Methyl jasmonate	49.68	–	–	0.35 ± 0.02	0.05 ± 0.00	Sweet, floral, jasmine a
46	Pentadecanoic acid, 14‐methyl‐, methyl ester	57.05	–	–	0.38 ± 0.05	0.35 ± 0.02	n.d.^c^
47	Palmitic acid, methyl ester	57.13	0.95 ± 0.05	0.91 ± 0.03	–	–	n.d.^c^
Alkenes							
48	Sabinene	40.52	–	–	0.25 ± 0.06	0.19 ± 0.07	Pepper, turpentine, wood^a^
49	*β*‐Caryophyllene	40.88	–	–	0.17 ± 0.04	0.15 ± 0.02	Wood^a^
50	*β*‐Cedrene	41.14	0.51 ± 0.06	0.44 ± 0.03	0.11 ± 0.02	0.22 ± 0.03	Woody^a^
51	*δ*－Cadinene	44.89	–	–	0.63 ± 0.08	0.46 ± 0.03	Thyme, medicine, wood a
52	*L*‐Calamenene	45.03			0.26 ± 0.05	0.16 ± 0.03	Herb, spice^a^
Hydrocarbons							
53	Tetradecane	46.59	1.66 ± 0.07	1.22 ± 0.08	0.67 ± 0.04	0.47 ± 0.05	Alkane^a^
54	Hexadecane	48.04	0.47 ± 0.05	0.75 ± 0.03	0.92 ± 0.02	0.62 ± 0.03	Alkane^a^
55	Heptadecane	51.97	0.86 ± 0.09	0.77 ± 0.08	0.51 ± 0.04	0.36 ± 0.03	Alkane^a^
56	2,6,10‐Trimethyltetradecane	52.06	1.63 ± 0.08	1.46 ± 0.09	0.95 ± 0.05	0.61 ± 0.07	Alkane^a^
57	Octadecane	54.64	1.96 ± 0.15	0.45 ± 0.03	0.39 ± 0.02	0.32 ± 0.02	Alkane^a^
Acids							
58	Octanoic acid	29.24	0.97 ± 0.04	0.98 ± 0.05	0.38 ± 0.03	‐	n.d.^c^
59	hexadecanoic acid	57.30	2.15 ± 0.21	4.19 ± 0.25	1.63 ± 0.10	1.20 ± 0.09	n.d.^c^
Phenolic compounds							
60	4‐Vinylphenol	35.73	0.33 ± 0.02	2.69 ± 0.08	0.15 ± 0.02	0.29 ± 0.03	Almond shell^a^
61	2,6‐Di‐tert‐butyl‐4‐methyl phenol	44.22	0.81 ± 0.07	1.16 ± 0.11	1.12 ± 0.07	0.26 ± 0.03	n.d.^c^
Other compounds							
62	Naphthalene	29.90	0.30 ± 0.02	0.64 ± 0.10	0.77 ± 0.05	0.53 ± 0.06	tar^a^
63	Indole	34.92	–	–	0.28 ± 0.01	0.59 ± 0.03	Mothball, burnt^a^
64	1‐Methy‐lnaphthalene	35.97	–	–	0.57 ± 0.06	0.50 ± 0.02	n.d.^c^
65	1,6‐Dimethylnaphthalene	35.41	–	–	0.30 ± 0.02	0.29 ± 0.02	n.d.^c^
66	Elemicin	45.77	–	2.01 ± 0.03	–	–	Spice, flower^a^

Note

Values showed here are average of three duplicates ± *SD*. “–” means undetected.

a http://www.flavornet.org/flavornet.html

b Du et al. (2014) (details see the References).

c not defined.

d http://wenku.baidu.com/view/1e9baa57f01dc281e53af04d.html

e Lv et al. (2012) (details see the References).

f https://www.chemicalbook.com/ChemicalProductProperty_CN_CB0251161.htm

**Figure 2:**
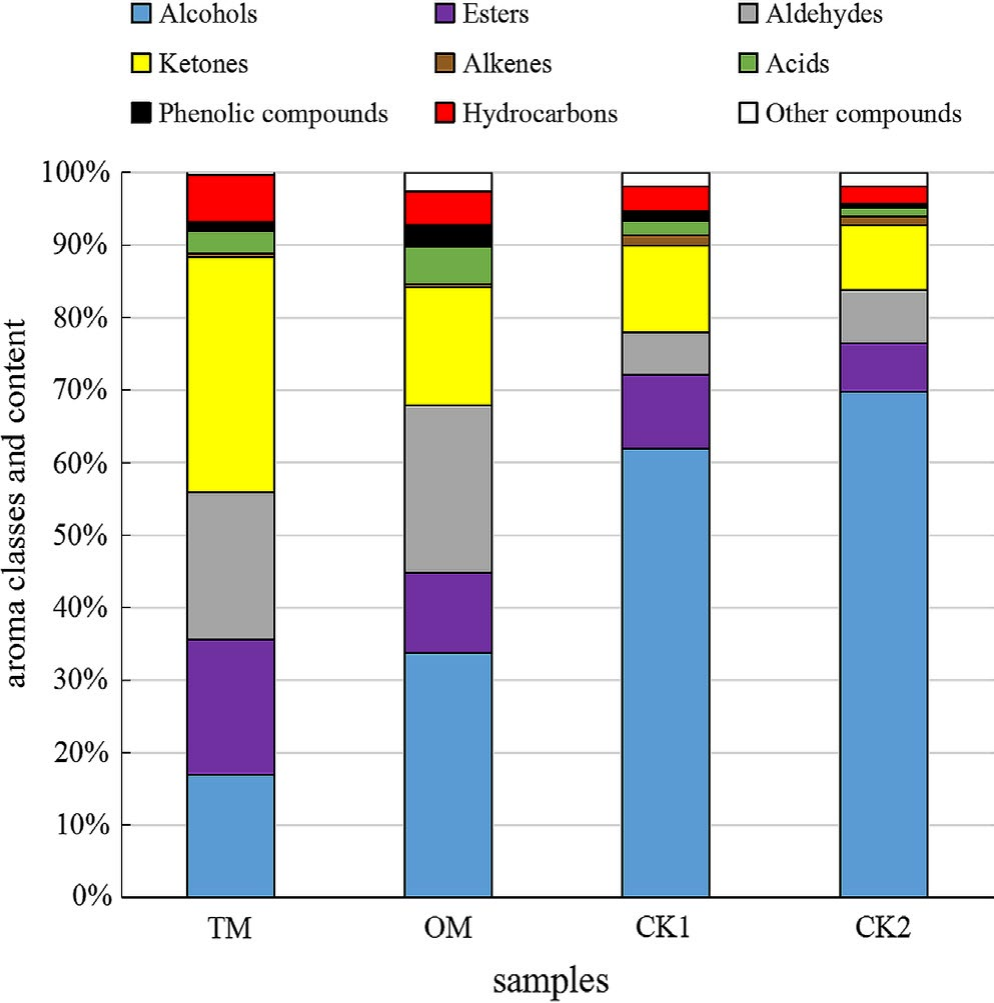
Classes of aroma compounds and content in mistletoes and CKs. Alcohols; Esters; Aldehydes; Ketones; Alkenes; Acids; Phenolic compounds; Hydrocarbons; Other compounds

**Figure 3:**
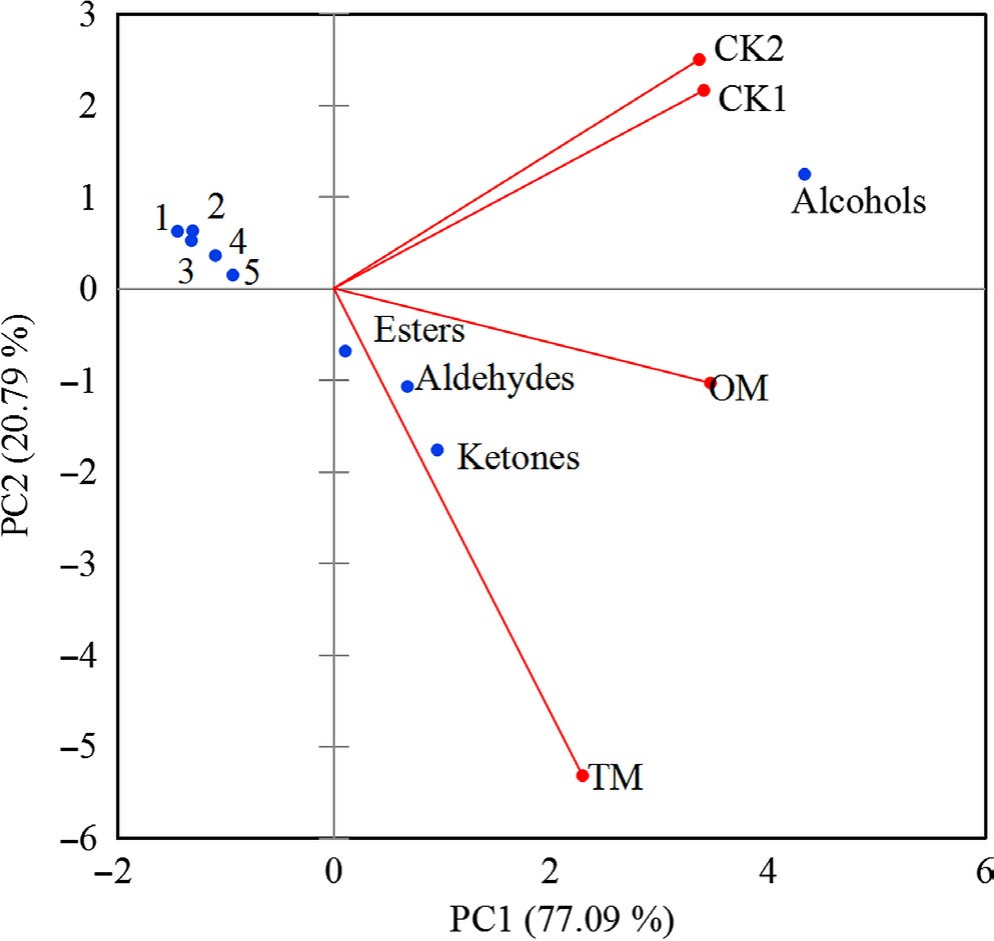
Principle component analysis (PCA) of the aroma classes in mistletoes and CKs by XLSTAT. 1. Alkenes; 2. Other compounds; 3. Phenolic compounds; 4. Acids; 5. Hydrocarbons

Aroma classes and contents were shown in Figure 2. It was showed that ketones were the most abundant aroma class in TM, and was significantly different from other aroma classes (*p* < .05). The alcohols in TM were significantly lower than CKs, in which the alcohols were the most abundant aroma classes, accounting for more than 60% of total aroma. However, the ketones, esters, and aldehydes content in the CKs were significantly lower than TM (*p* < .05). Figure 3 showed the PCA results of the aroma classes in different samples. The first two principal components (PCs) accounted for 97.88% of the variation in the data. The PC1 dimension, which explained 77.09% of the variance, showed that samples were mainly characterized by ketones, alcohols, alkenes, and phenolic compounds, etc. The PC2 dimension explained an additional 20.79% of the variance, characterized by ketones, alcohols, and aldehydes. The positive PC1 and negative PC2 dimension was largely correlated with ketones and aldehydes, which were main aroma classes in mistletoes. The positive PC1 and PC2 were correlated with the alcohols, the dominant aroma class in CKs. It could be deduced that the mistleotes were differentiated from CKs due to their different aroma classes and material bases. Basically, the result of PCA agreed with that of Figure 3.

A large number of ketone compounds, including *β‐*ionone, nonanone, geranylacetone, *α*‐ionone, epoxy‐*β*‐ionone, 2‐decanone, 2‐undecanone, nonanone, etc., were detected in this research and imparted fruit, sweet, fresh, and wood characters. It was found that geranylacetone and epoxy‐*β*‐ionone (7.92% and 7.71%) were the most abundant ketone compounds in TM and produced fruity and floral odors, significantly greater than other samples (*p* < .05). Epoxy‐*β*‐ionone, a degradation product of *β*‐carotene (Ravichandran, 2002), contributed to the floral and sweet scents in TM. *β*‐Ionone had violet and woody odors and a very low odor threshold for human detection (Schuh & Schieberle, 2006) and was usually found in black tea with high amounts (Rawat et al., 2007), and was an important contribution to tea aroma.

Nine alcohols, including *β*‐linalool, phenethyl alcohol, hotrienol, geraniol, *α*‐cedrol, *cis*, linalool oxide, *α*‐terpineol, *E*‐nerolidol, and *β*‐ionol, were detected in TM. Among them, *β*‐linalool was in a high content in the mistletoes, accounting for 7.35% and 10.41% of the total aromas in TM and OM, respectively, significantly lower than the CKs (*p* < .05). It was because that the CKs belonged to large tea leaf of Yunnan varieties. Previous research showed that black teas made from large tea leaf of Yunnan varieties contained high content of *β*‐linalool (Wang et al., 2012). *β*‐Linalool was one of the terpene alcohols in teas aroma, endowing wood, sweet, and floral notes to tea (Rawat et al., 2007). The high concentration and low threshold level made it a great contribution to the total tea flavor (Ravichandran, 2004). Our results were consistent with the previous studies in that *β*‐linalool was the principal alcohol in green tea (Kato & Shibamoto, 2001; Tontul et al., 2013) and black tea (Wang et al., 2012). The phenethyl alcohol content in OM (13.08%) was significantly greater than other samples, which imparted sweet and floral aromas. Hotrienol, with hyacinthine odor, rarely reported in tea aroma, was detected in all samples with high content.

Aldehydes were detected from mistletoes and included benzaldehyde, (*E*)‐2‐decenal *Z‐*2‐decenal, *β*‐cyclocitral, safranal, benzyl acetaldehyde, etc. Aldehydes content in mistletoes was significantly higher than CKs (*p* < .05). Benzaldehyde was an important active odor in almonds and nuts, contributing significantly to the almond odor (Lasekan & Abbas, 2010; Xiao et al., 2014). Benzaldehyde was rarely found in as high of an amount in teas as in mistletoes, which was a key difference from teas. Aldehydes such as 2‐heptenal and pentanal mainly came from oxidative degradation of amino acids and fatty acids (Rizzo, 2014), and they were important VFCs due to their low odor threshold (Selli & Cayhan, 2009).

Esters detected in this study included methyl salicylate, *cis*‐3‐hexenyl isovalerate, nerol acetate, dihydroactinidiolide, *γ‐*nonanolide, *cis*‐hexanoic acid‐3‐hexenyl ester, etc. The total amount of esters in the mistletoes and CKs were significantly different. Among the esters, methyl salicylate, contributing floral, fruity, and sweet characters (Katsuno et al., 2014), was frequently detected in black tea with high amount and provided a strong mint scent (Rawat et al., 2007). *γ*‐Nonanolide was detected as one of the esters only in the mistletoes and was rarely found in teas, which imparted strong coconut flavor with 0.0097 mg/m^3^ aroma threshold in water (Van Gemert, 2011). Our results suggested that *γ*‐nonanolide was one of the major differences between TM and CKs in aroma compounds. Methyl jasmonate, releasing jasmine‐like odor and often found in teas (Mu et al., 2018), was also identified with a low aroma intensity in this study.

Five kinds of hydrocarbons were identified with GC–MS, constituting 6.58% and 4.65% of the total aroma in TM and OM, respectively. Researches had found that few odors came from hydrocarbons (Du et al., 2014; Lv et al., 2012) and thus they had little effect on tea aroma. Alkenes, such as sabinene, *β*‐caryophyllene, *β*‐cedrene, *δ*‐cadinene, and *L*‐calamenene, were found to have an important contribution to the woody or herbal flavor in tea (Du et al., 2014; Lv et al., 2012). However, only *β*‐Cedrene was detected in mistletoes in a low content.

Two kinds of acids, hexadecanoic acid and octanoic acid, were detected in this study. The result was different from the findings of Du et al., (2014), who did not detect hexadecanoic acid in Pu‐erh tea by SPME and SDE methods. This discrepancy might be attributed to different experimental conditions and samples.

Two phenolic compounds, 2, 6‐di‐tert‐butyl‐4‐methyl phenol and 4‐vinylphenol, did not account for a significant proportion in the total VFCs in this study. Indole was detected in this study, which was an important compound in the formation of aroma in oolong teas (Zeng et al., 2016). The elemicin was only found in OM, providing spice and flower odors.

### 3.2 Principal components analysis (PCA)

**Figure 4:**
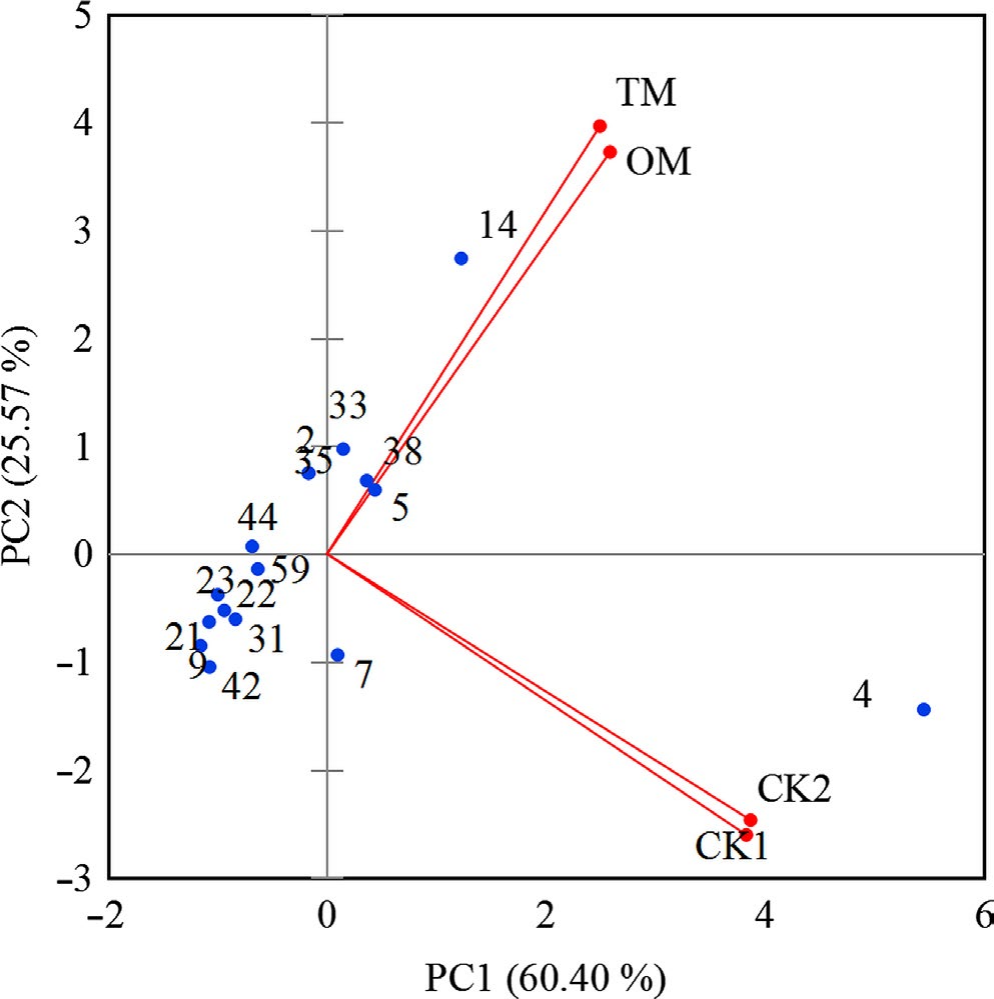
Principle component analysis (PCA) of the aroma components in mistletoes and CKs by XLSTAT. Aroma compounds were treated before PCA. 2: Phenylethyl alcohol; 4: *β*‐Linalool, 5: Hotrienol, 7: Geraniol; 9: *E*‐Nerolidol; 14: Benzaldehyde; 21: Decanal; 23: (*E*)‐2‐Decenal; 31: *α*‐Ionone; 33: Geranylacetone; 35: Epoxy‐*β*‐ionone; 38: Methyl Salicylate; 42: *cis*‐Hexanoic acid, 3‐hexenyl ester; 44: Dihydro actinidiolide

Before PCA, data in Table 3 were processed as follows. The hydrocarbons and aromas whose content was <1.0% were removed from the list; the remaining aroma substances were subjected to PCA (Figure 4). The first two PCs accounted for 85.97% of the variation of the aroma compounds. The PC1 dimension (60.4% of the variance) showed that it was mainly characterized by *β*‐linalool, benzaldehyde, (*E*)‐2‐decenal, *α*‐ionone, phenethyl alcohol, *Z*‐decanal and (*E*)‐nerolidol, *cis*‐hexanoic acid, 3‐hexenyl ester, etc. The PC2 dimension explained an additional 25.57% of the variance, which was largely correlated with *β*‐linalool, benzaldehyde, *Z*‐decanal and geranylacetone, *cis*‐hexanoic acid, 3‐hexenyl ester, methyl salicylate, and geraniol, etc. The positive PC1 and PC2 dimensions were correlated with benzaldehyde, hotrienol, methyl salicylate, and geranylacetone, which were the dominant aroma compounds in TM and OM. For the positive PC1 dimension, it was largely correlated with *β*‐linalool. For the negative PC2 dimension, it was characterized by geraniol, *E*‐nerolidol, decanal and *cis*‐hexanoic acid, 3‐hexenyl ester. Figure 4 showed that TM could be differentiated from CKs due to their aroma compounds.

**Table 3:** Main nutritional constituents in experiment samples

Chemical components (% dry weight)	TM	OM	CK1	CK2
Soluble water extract	35.12 ± 1.05^b^	30.28 ± 2.01^c^	40.15 ± 1.07^a^	42.39 ± 2.10^a^
Polyphenols	0.600 ± 0.07^c^	1.780 ± 0.05^b^	26.95 ± 0.72^a^	27.19 ± 1.31^a^
Amino acids	1.760 ± 0.17^b^	1.020 ± 0.12^c^	3.080 ± 0.22^a^	3.47 ± 0.20^a^
Carbohydrates	2.810 ± 0.12^d^	6.700 ± 0.11^a^	3.250 ± 0.21^c^	4.130 ± 0.14^b^
Caffeine	0.041 ± 0.01^b^	0.016 ± 0.01^c^	3.628 ± 0.25^a^	3.435 ± 0.21^a^
GC	0.133 ± 0.04^c^	0.043 ± 0.01^b^	0.949 ± 0.03^a^	0.929 ± 0.05^a^
EGC	0.033 ± 0.00^b^	0.032 ± 0.00^b^	1.865 ± 0.07^a^	1.681 ± 0.08^a^
C	0.176 ± 0.09^c^	0.009 ± 0.00^d^	1.924 ± 0.12^b^	2.309 ± 0.17^a^
EGCG	0.018 ± 0.00^c^	0.013 ± 0.00^c^	9.080 ± 0.47^a^	5.472 ± 0.23^b^
EC	0.024 ± 0.01^c^	0.035 ± 0.00^c^	0.912 ± 0.04^b^	2.070 ± 0.19^a^
GCG	0.018 ± 0.00^d^	0.032 ± 0.00^c^	0.141 ± 0.01^a^	0.117 ± 0.01^b^
ECG	0.016 ± 0.00^c^	0.662 ± 0.01^a^	0.013 ± 0.00^c^	0.055 ± 0.01^b^
CG	0.007 ± 0.00^d^	0.380 ± 0.01^c^	4.250 ± 0.26^b^	7.552 ± 0.29^a^

Note

Data are means (±*SD*) of 3 replicates. The different upper lowercase means different in the treatments.

^a,b,c,d^Different letters in the same row indicate significant differences between mean values (*p* < .05).

**Figure 5:**
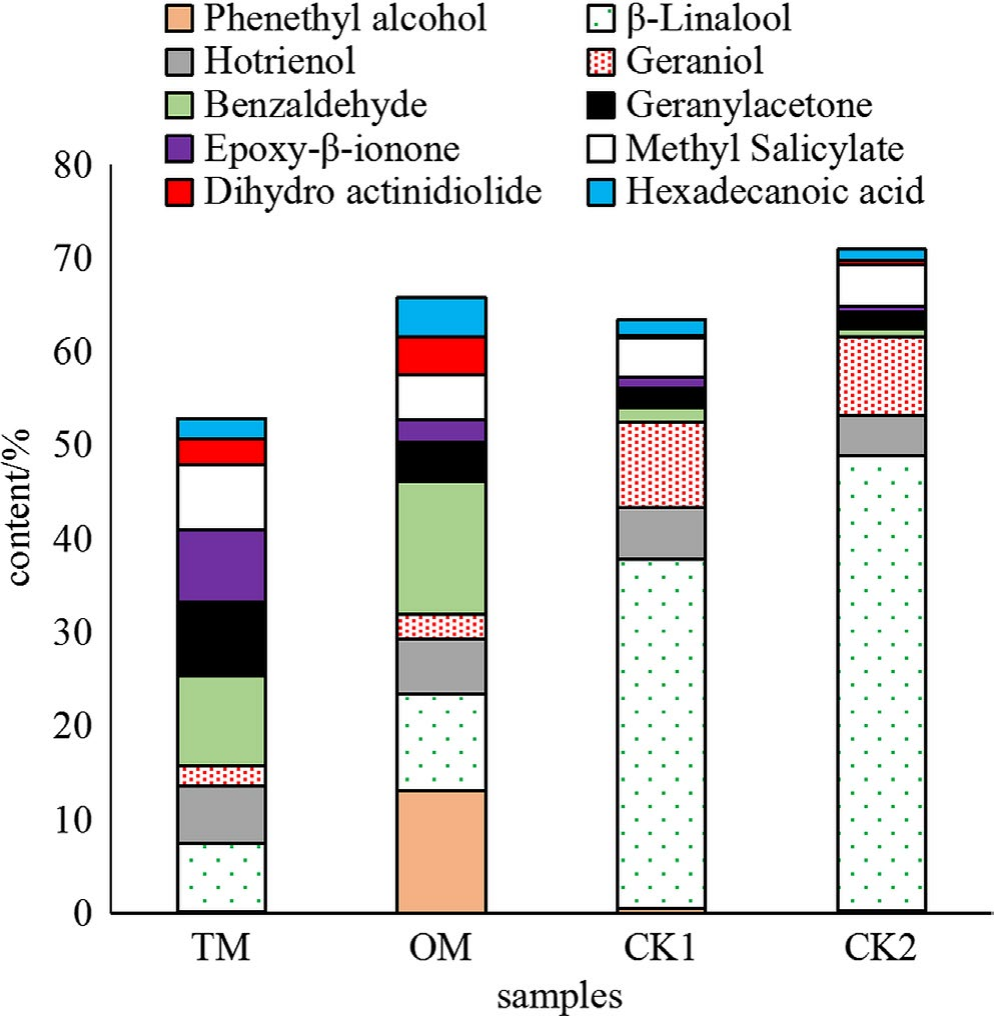
Dominate aroma components in mistletoes and CKs. Phenethy alcohol; *β*‐Linalool; Hotrienol; Geraniol; Benzaldehyde; Geranylacetone; Epoxy‐*β*‐ionone; Methyl Salicylate; Dihydro actinidiolide; Hexadecanoic Acid

The major aroma components were significantly different in contents between CKs and TM (Figure 5). It was evident that benzaldehyde, *β*‐linalool, methyl salicylate, geranylaceone, and epoxy‐*β*‐ionone were important aroma components in TM. In OM, benzaldehyde, phenethyl alcohol, and *β*‐linalool were principal aroma compounds. *β*‐ Linalool and geraniol were most abundant aroma compounds in CK1 and CK2 because of their large leaf origins.

### 3.3 Aggregation of aroma compounds with different odor notes

**Figure 6:**
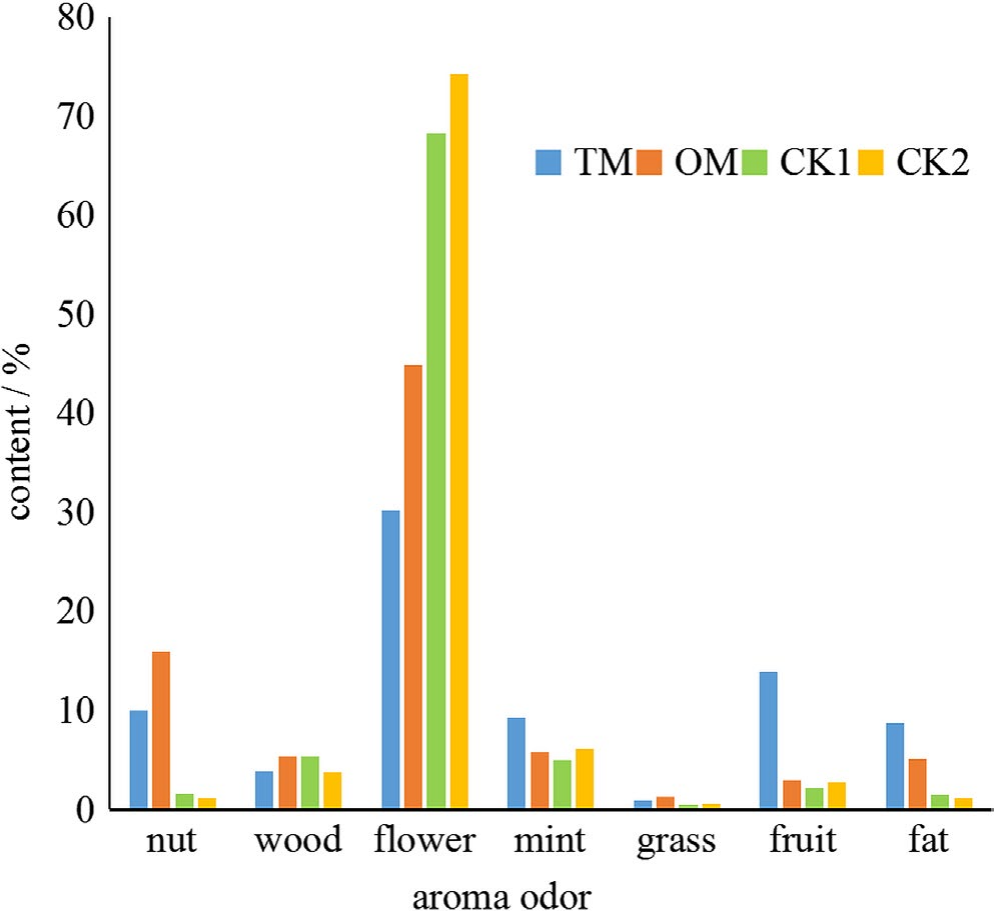
Summary of aroma compounds with different odor notes. TM; OM; CK1; CK2

According to the odor of different components (Table 2), compounds with flower, fruit, mint, wood, nut, grass, and fat odor were summarized in Figure 6. It was evident that the CKs contained more floral odor compounds than TM and OM. The nut flavor compounds in TM and OM were significantly higher than that in the CKs, mainly due to the benzaldehyde. It was worth noting that content of different aroma compounds with different odor note only represented the quantity in samples but not the real aroma smelt by human noses. The threshold and content of each aroma compound both decided the aroma characteristics.

It was known that SPME/GC–MS method had drawbacks in identification of odor intensity, and the main contributors of flavors were usually analyzed by Gas Chromatography Olfactometry (GC–O) or Aroma Extract Dilution Analysis (AEDA) methods, which were widely used in aroma researches (Mahmoud & Buettner, 2016; Pham et al., 2008). However, due to the limitation of experimental conditions, GC–O and AEDA were not conducted in this research. In future research, GC–O and AEDA can be chosen to analyze the main contributors of flavors in teas.

### 3.4 Nutritional components analysis

#### 3.4.1 The total soluble solids

To investigate the differences in nutritional qualities between mistletoes and teas, the quality index was determined in this research. The contents of nutritional components were found different between mistletoes and tea samples (Table 3). The total soluble solids were 35.12% in TM, significant lower than the CKs. It was because that TM did not experience rolling process like tea leaves, and its tissues and cells were not broken, which led to less releasing of the soluble solids. The total soluble solids represented the thickness of the infusion, and the higher soluble solids would lead to the mellow and smooth mouth taste. In sensory evaluation, the taste of TM was not as mellow and smooth as CK2 (Table 4).

**Table 4:** Sensory quality and scores of the mistletoes and CKs

Sample name	Total scores	Outer shape		Color of infusion		Aroma of infusion		Taste of infusion		Residue of sample	
		Comments	Score	Comments	Score	Comments	Score	Comments	Score	Comments	Score
TM	85.55	Short crab leg, smooth, even, green	87	Bright yellow and green	84	Fresh, lasting, nutty and woody aroma with minor floral scent	90	Thick, mellow, smooth	83	Hard, even, bright	80
OM	84.80	Long crab leg, not even, vertical wrinkle, yellowish green	86	Light yellow	89	Clean, nutty and woody with grassy scent	88	Plain, sweet after taste	82	Hard, even	78
CK1	87.30	Light and loose, dark green, even	88	Bright, light green	86	Clean and lasting, fresh aroma	92	Fresh, astringent	84	Soft, even, bright	85
CK2	90.40	Tight and heavy, green, even and neat	91	Bright, greenish yellow	90	Lasting and high, pure and fresh	93	Mellow and brisk, Sweet after taste	90	even, bright	84

The total contents of polyphenols, amino acids, carbohydrates, and caffeine in TM were significantly lower than the total soluble solids, which meant that there were still lots of components undetected and needed to be analyzed further. Previous researches showed that mistletoes contained more functional flavonoids, sesquiterpene lactones, lectins, steroids, organic acids (small molecules), peptides, proteins, polysaccharides (polymers), and alkaloids (Harikrishnana, Balasundaramb, & Heo, 2011; Ni et al., 2013; Samuelsson, 1958). Therefore, mistletoes could be used as a source of functional plant resources to extract more health beneficial substances.

#### 3.4.2 Tea polyphenols, catechins, and caffeine

Polyphenols are important factors in tea qualities (Wu et al., 2019; Xu et al., 2018), which contribute astringency to the tea taste. The catechins are the family of polyphenols, mainly include EC, EGC, ECG, EGCG, C, GC, CG, and GCG and have been used as a large group of natural antioxidants in many aspects (Singh, Shankar, & Srivastava, 2011; Wang, Dong, Men, Tong, & Zhou, 2013). Tea catechins can be categorized into two groups: nonesterified and esterified catechins (Theppakorn, 2016). Nonesterified catechins include C, EC, GC, and EGC; whereas the esterified catechins contain EGCG, ECG, GCG, and CG (El‐Shahawi, Hamza, BahaffiA, Al‐Sibaai, & Abduljabbar, 2012; Theppakorn, 2016). Table 3 showed that the contents of polyphenols and catechins in TM were at trace levels, significantly different from CKs (*p* < .05). The CKs were green teas in nature, and therefore, they were rich in polyphenols and catechins (Wei et al., 2011). Nonesterified catechins were significantly different from the esterified catechins in TM. However, there was an opposite tendency in CKs. EGCG was the principal catechin with a high content in CKs, which was in agreement with the reports of Khan and Mukhtar (2007) and Zuo, Chen, and Deng (2002). The results also showed that the content of caffeine in TM was quite low, significantly different from CKs (*p* < .05). Caffeine contents in CKs were between 3.435% and 3.628% of the dry weight, which agreed with the findings of Villanueva Bermejo et al. (2015).

#### 3.4.3 Amino acids and soluble carbohydrates

Amino acids and soluble carbohydrate contents in TM were in a moderate level, compared with polyphenols, catechins, and caffeine. The amino acids and soluble carbohydrates were important contributor to the taste of tea soup, representing umami and sweetness, respectively. The low content of amino acids in TM made it not as umami as CKs. It was worth mentioning that the soluble carbohydrate contents in OM were higher than in other samples, which made it taste sweeter. It was reported that mistletoe widely distributed both in Europe and Asia contained lots of polysaccharides (Harikrishnana et al., 2011), which agreed with our results.

Our results showed that the nutritional compositions in mistletoes were quite different from tea samples, indicating that the mistletoes did not absorb large quantities of chemicals from tea trees as a source of nutrition despite they were parasitic on tea trees. Therefore, the TM should have its own material circulation and metabolism, which needs to be further studied in the future.

The theaflavins, the index of redness in black tea soup, were not determined in this research because they were not important quality parameters in mistletoe, green teas, and Pu‐erh teas (Okinda Owuor et al., 2006).

### 3.5 Sensory evaluation

Sensory evaluation was carried out by five experienced tea experts from our research institute. The mistletoes were evaluated according to sensory test method of green teas because of their unfermented nature. Sensory scores of the mistletoes and CKs ranged from 84.80 to 90.40, according to the outer shape, the color, aroma, and taste of the infusion, the residues (Table 4). Fresh, lasting, woody, and nutty aroma was the characteristic of TM. TM had its own sensory attributes, significantly different from teas. Despite the lower scores, the panelists believed that the two mistletoes were acceptable both in aroma and taste, which could be further developed into alternative tea drinks.

## 4 CONCLUSIONS

Sixty‐six volatiles were identified in research samples, including alcohols, aldehydes, ketones, esters, hydrocarbons, acids, phenolic compounds, and alkenes. According to PCA, ketones, alcohols, and aldehydes were the principal aroma classes in TM. Benzaldehyde (9.64%), epoxy‐*β*‐ionone (7.71%), geranylacetone (7.92%), *β*‐linalool (7.35%), and methyl salicylate (6.96%) were the most abundant and significant components in TM, which contributed to a fresh, lasting woody, and nutty aroma with minor floral scent. The mistletoes could be differentiated from CKs due to the difference of the aroma compounds according to PCA.

Analysis of nutritional components showed that a large portion of compositions in teas such as the polyphenols, catechins, caffeine, free amino acids were relatively low in TM, indicating there were still ingredients undetected in the water extract. This research also testified that the TM did not absorb large quantities of chemicals from tea tree as a source of nutrition despite it being parasitic on tea trees. Sensory test showed that the TM and OM had acceptable aroma and taste, which could be developed into alternative tea drinks in the future. This study sheds light on the volatile and nutritional qualities in the mistletoes and will provide a scientific foundation for their further utilization.

## CONFLICT OF INTEREST

The authors declare that they do not have any conflict of interest.

## ETHICAL APPROVAL

This study does not involve any human or animal testing. Written informed consent was obtained from all study participants.
